# Tuberculosis in Hematopoietic Stem Cell Transplant Recipients

**DOI:** 10.4084/MJHID.2013.061

**Published:** 2013-11-04

**Authors:** Jéssica Fernandes Ramos, Marjorie Vieira Batista, Silvia Figueiredo Costa

**Affiliations:** 1 Group of Infection in immunocompromised patients of Hospital das Clinicas of University of São Paulo, Brazil.; 2 Department of Infectious Diseases, Medical School, University of São Paulo, São Paulo, Brazil.

## Abstract

Literature on tuberculosis (TB) occurring in recipients of Hematopoietic Stem Cell Transplant (HSCT) is scanty even in countries where TB is common. Most reports of TB in HSCT patients were from ASIA, in fact the TB incidence ranging from 0.0014 (USA) to 16% (Pakistan). There are few reports of TB diagnosis during the first two weeks after HSCT; most of cases described in the literature occurred after 90 days of HSCT, and the lung was the organ most involved. The mortality ranged from 0 to 50% and is higher in allogeneic HSCT than in autologous. There is no consensus regarding the screening with tuberculin skin test or QuantiFERON-TB gold, primary prophylaxis for latent TB, and whether the epidemiologic query should be emphasized in developing countries with high prevalence of TB.

## Introduction

Hematopoietic Stem cell transplant (HSCT) recipients have severe impairment in cell-mediated immunity as result of the conditioning regimen, immunosuppressive therapy and graft-versus-host disease (GVHD).^1^,^2^ Accordingly, they are susceptible to bacterial, viral, and fungal infections. Mycobacterial infections can also occur in these patients, although the incidence is not high, even in countries where tuberculosis (TB) is common.^2^ TB in this population of patient is mainly due to reactivation of latent infection.^2^ However, in country where TB is endemic, pulmonary tuberculosis could be due to a new infection.

Data from the United States showed that the incidence of mycobacterium infection (MBI) among HSCT ranges from 0, 0014 % to 3%.^1^,^3^,^4^,^5^ Countries in which the prevalence of tuberculosis in the general population is higher than it is in the United States have reported incidences varying from 1.6% in Spain^6^ and Turkey,^7^ 8.57% in Hong Kong and Taiwan to 16% in Pakistan.^8^–^11^

There are few reports of TB diagnosis during the first two weeks after HSCT (Table 1). Most of cases described in the literature occurred after 90 days of HSCT.^12^–^28^

The incidence of TB in HSCT ranged from 0, 0014% to 16% (Table 1). The diagnosis of TB varied from +21 to +1410 (median of 257, 2 days) after the HSCT. The lung was the organ most often involved. Most of cases were described in allogeneic HSCT, and the mortality in this type of HSCT was high, varying from to 0% to 50% (Table 1).

There is no consensus regarding the screening with tuberculin skin test or QuantiFERON-TB gold and primary prophylaxis for latent TB.^29^–^33^

## TB Risk Factors

The increased risk of tuberculosis in this setting could be explained by severely impaired cell-mediated immunity as a result of their underlying illness and conditioning chemotherapy. However, there is a lack of prospective studies on TB in HSCT and the clinical, radiological and epidemiological data are based on case reports and retrospective analysis with a small number of patients.^27^,^34^,^35^,^37^ Previous study had showed the main risk factors for tuberculosis, comparing to control group, were allogeneic transplantations with unrelated donors and total body irradiation (TBI), with a relative risk (RR) of 23.9 and 4.9 respectively (p<0.05), and chronic GVHD with RR of 3.6 (p<0.05).^34^ Another retrospective study observed that the majority of allogeneic patients with post-transplant tuberculosis received corticosteroids for GVHD.^27^ Patients with chronic GVHD have a marked delay of T cell subset recovery with low numbers of CD4 cells being present. As this period of impairment of T cell function may be indefinite in the presence of chronic GVHD, it might contribute to an increased predisposition to tuberculosis.^35^,^36^,^37^

## Symptoms of TB diseases

The clinical presentation of tuberculosis in patients undergoing HSCT may have a wide range of signs, symptoms and radiological findings (Table 1). Tuberculosis following conventional HSCT usually presents indolent clinical courses. The lung is the most common site of the disease, and the usual manifestations are fever, cough, dyspnea and hypoxemia (Table 1). Although the clinical and radiographic presentation of TB in the HSCT population usually mimics that in the non-transplant population, atypical presentations have been described, such as diffuse alveolar hemorrhage.^34^ In immunocompromised host patients, the radiological finding could be mitigated or absent on chest x-ray and on this panel the CT-scan provided additional information to help diagnose pulmonary TB.^38^

In regard to the radiologic features of pulmonary tuberculosis in HSCT recipients, recent studies evaluated chest X-ray^10^ and CT-scan.^7^ The chest X-ray abnormalities were air-space consolidation (100%) and nodules (80%). On chest CT scans, the most common parenchymal lesions were consolidation (100%), nodules (71%), tree-in-bud appearance (43%), and ground-glass opacity (43%). Cavities were present on CT scans in only 14% of the study patients and lymphadenopathies weres noted in 71%.

## TB Diagnosis

**A** suggestive clinical and radiologic finding is not enough to allow initiating specific treatment in patients submitted to HSCT, as recommended in populations of high endemic TB countries. The bacteriological diagnosis is crucial once the incidence is low and differential etiology should be excluded.

Similarly to other scenarios, the main challenge is to obtain a result as fast as possible to start appropriate treatment and establish environmental infection control measures.^2^

Thus, the recommendation is to detect the presence of acid-fast bacilli (AFB) in samples as soon as possible and that these samples should be cultured for identification and antimicrobial susceptibility testing.^39^ Some authors had already reported cases of transplanted patients with disseminated non-tuberculosis mycobacteria infection and drug resistant TB, reinforcing the importance of such complimentary tests.^40^

An important obstacle to a fast invasive diagnostic test is the clinical condition characterized by bone marrow suppression (anemia, thrombocytopenia), although some studies showed that investigational procedures like lung CT guide-biopsy are efficacious and safe in this population.^41^

A recently published review of 56 cases of tuberculosis in HSCT, showed that only 55% of cases were diagnosed with culture, whereas acid-fast bacilli (26%) was the second most common approach for diagnosis and histology was responsible for 20,3%.^2^

Molecular methods are increasingly used in diagnosis since they are faster than mycobacterial cultures (3–6 weeks) and have the potential role to determine species. However, there are still some issues in interpreting mycobacterial DNA in respiratory samples (Table 2).^43^,^44^,^46^ Lee et al^45^ studied real time PCR in CT guided BAL in 99 patients unable to produce sputum samples or with AFB negative result: PCR showed a positive predictive value in 81% out of 27 patients with confirmed TB, although with a lower sensitivity.

In general population molecular tests are increasingly used, and several guidelines as UK most recent recommendations (2011) from the National Institute for Health and Clinical Excellence (NICE) on the diagnosis of latent tuberculosis and of active tuberculosis even mention these methods.^47^

The common approach is to use rapid diagnostic tests for *Mycobacterium tuberculosis* complex (*M tuberculosis*, *M bovis*, *M africanum*) on bronchial specimens of patients only if rapid confirmation would alter the patient’s care or before conducting a large contact tracing initiative. Such tests are not recommended for pleural, cerebrospinal fluid, or urine in order to exclude the diagnosis of TB as they have a high false negative rate.^48^

The Xpert MTB/RIF assay, which enables simultaneous detection of *Mycobacterium tuberculosis* (MTB) and rifampicin (RIF) resistance, was endorsed by World Health Organization (WHO) in December, 2010. This assay was specifically recommended for use as the initial diagnostic test for suspected drug-resistant or HIV-associated pulmonary tuberculosis. One Xpert MTB/RIF test on sputum detects 90% of pulmonary tuberculosis (99% of smear-positive disease and about 75% of smear-negative disease). Such high sensitivity of Xpert MTB/RIF for rifampicin resistance is accompanied by some false-positive results and confirmatory drug sensitivity testing is needed.^49^

A different test based on antigen detection was already studied in the general population. Rajpal and colleagues studied an India tribal population where nearly 80% suffers from malnutrition. In this group, they compared 41 patients with active TB (90% confirmed with AFB and/or culture positive) with 87 controls to three different diagnostic blood tests: ELISA based TB antigen, ADA and in house IS6110 based PCR. The positivity of PCR assay in TB patients was around 48.78%, which was found to be significant (p < 0.0001) than the control group (2.30%). Mean absorbance of Antigen 85 ELISA in TB group was significantly (p<0.05) higher than non-TB control group. There was no immunocompromised patient (HIV or immunossupressors) in their cohort.^50^

Blood TB PCR is still not standardized, but offers great promise for the rapid and specific diagnosis. Indian investigators recently evaluated a multiplex nested PCR method targeting insertion sequence 6110 and MPB64 sequence in 130 samples. The sensitivity and specificity of the assay was respectively 95.7% and 100% with a negative predictive value of 99.2%.^51^

Molecular techniques such as polymerase chain reaction (PCR) was rarely used for diagnosis in HSCT patients reported (3.7%), and are mainly used in combination with other diagnostic approach.^2^

There are few studies, however, comparing accuracy of different tests in this transplant setting, and most of the data come from general population, mainly from pulmonary TB.^46^,^57^

## Treatment of TB

The use of drugs against TB should be in agreement with local recommendations, based on surveillance of mycobacterial resistance patterns to antimicrobial. Since the first drugs available in 1940s, the subsequent emergence of resistant species led to combination therapy.^53^,^54^ In fact the treatment of TB in HSCT recipients are similar to the general population with two important differences: the duration of therapy, normally longer, and the drug regimen, because interaction was reported between rifamycins (rifampicin and rifabutin) and immunosuppressive drugs like cyclosporine.

The approach recommended by World Health Organization, North American and European guidelines is a 6-month chemotherapy regimen using a combination of 4 drugs (rifampicin, isoniazid, ethambutol, and pyrazinamide for 2 months, followed by rifampicin and isoniazid for 4 months) with cure rates of approximately 90% of drug-susceptible cases.^54^,^55^,^56^ The Brazilian recommendation is in agreement with other countries (Table 3).^57^

Most of the reports with combination therapy in HSCT patients had shown good results. La Camara and colleagues treated nineteen patients with a median of three drugs. Seventeen received more than 3 months of treatment and all were cured. An interesting finding of this study was the interaction between cyclosporine with rifampicin, with three patients presenting a decrease in cyclosporine levels, two of whom with worsening in chronic GVHD. Three patients (18%) developed hepatotoxicity.^56^

In more pronounced immunosuppression state like in unrelated cord blood transplantation there is already TB cases reported^52^,^53^ In this cases the therapy of choice, even with possible drug interaction, was the classic four drugs of therapeutic guidelines.

The treatment of tuberculosis (TB) is complicated by drug-induced hepatotoxicity, one of the leading types of drug-induced liver injury (DILI). Current recommendations are to obtain baseline biochemical testing in all patients being treated for TB, but there is a lack of evidence, these recommendations are based on observational studies and expert opinion. It is important to notice the phenomenon of adaptation (i.e., temporary stress or mild injury to the liver) that occurs in 20% or more of patients treated with anti-TB medications and often results in cessation or interruption of treatment.^62^

Some authors advocate that concerted efforts at identifying biomarkers or hepatic enzymes trending to prevent severe DILI.^63^

Another issue of therapy is the interaction between rifampicin and immunosuppressive drugs used in GVHD prophylaxis. Rifampicin reduces the serum levels of cyclosporine and sirolimus.^56^ Rifabutin could be an alternative, because it is a weaker inducer of cytochrome P450, but the clinical experience is limited with just one report.^57^ It is common in most of treatment descriptions tapering of cyclosporine and temporary addition of corticosteroids.

In spite of some prescribing practices and poor patient compliance had increased the risk of selection of drug-resistant (DR) strains of Mycobacterium tuberculosis, which are more difficult and more expensive to treat, reports of tuberculosis resistant in HSCT are rare. Cordonnier and colleagues reported 20 cases of tuberculosis. Susceptibility test results were available in 12 cases and none was resistant to any drug tested.^11^ Nevertheless a case of MDR-TB (which is resistance to rifampicin and isoniazid) have already been published in this population and was successfully treated with **c**ycloserine 500mg, levofloxacin 500mg, PAS 10g and kanamycin 1g three times a week during 18 months, with no relapse.^64^

Fortunately a portfolio of promising new TB drugs is on the horizon. Eleven new or repurposed tuberculosis drugs are in clinical investigation, 3 in phase 3 trials, which are evaluating the possibility to shorten treatment to 4 months, using strategies like inclusion of a third-generation fluoroquinolone or rifapentine (a semisynthetic rifamycin that has a longer half-life than rifampicin) and compounds as linezolid, probably a limited option in HSCT patients due the myelotoxicity when used for long period.^65^

Some perspectives in TB treatment with adjunct immunotherapies should require caution in HSCT recipients, since the safety regarding GVHD or bone marrow rejection is not well studied.^66^

## TB Latent

In some countries the use of tests based on interferon γ release assays appear to be useful to determine latent TB infection, but further studies are needed to define their utility in immunocompromised host.^42^,^43^

TB screening using TST or QuantiFERON-TB gold (QFT-GIT) in HSCT is controversial.^29^,^30^,^31^,^32^ T cells play an important role in protective immunity against tuberculosis. The question arises as to whether TST-specific memory T cells are transferred from the marrow donor to the recipient and persist in the long-term.^12^

The Infectious Diseases Working Party of the European Blood and Marrow Transplant Group survey showed that 51% of centers had a program of vaccination against tuberculosis in their countries, only 10% systematically screening their patient before transplant by TST, and 51% screening in case of suspicious.^11^

A study of 295 HSCT in Korea showed an incidence of TB of 3.1%.^19^ Multivariate analysis revealed that only a previous history of TB infection and total Body Irradiation (TBI) increased the risk of TB infection in HSCT patients (relative risk, 4.8 and 12.5, respectively).^18^

Isoniazid prophylaxis in HSCT recipients with only radiological findings suggestive of past inactive TB infection did not significantly alter the incidence of TB infections (P = 0.236).^18^ Other study evaluated the frequency of tuberculin skin test (TST) positivity among 26 patients and their donors screened by TST to investigate whether tuberculin positivity of a recipient or donor influenced the rate of tuberculosis disease, transplant-related events, and to evaluate the effectiveness of isoniazid (INAH) prophylaxis administered to those with positive TST.^32^ The frequency of TST positivity was 23% (n = 6) among recipients and also 23% (n = 6) among donors. Two recipients and five donors with positive TST received INAH prophylaxis for six months. The transplantation procedure was not postponed for either recipient or donor TST positivity. Despite the high frequency of tuberculosis in their country, they have not detected any case of tuberculosis in their center, either among the TST screened (n = 26) or non-screened (n = 128).^32^

A recent study compared the simultaneous use of QFT-GIT and TST (>5 mm) and found similar frequencies of positive outcomes in the two screening test, although the overall agreement was poor (Kappa=0.08, 95% CI −0.006 to 0.24). However, the study did not have adequate power. The authors evaluated a cohort of 244 patients, 100 autologous and 144 allogeneic recipients during a 28-month period. No prophylaxis for latent tuberculosis was administrated, 201 (82%) patients had Bacillus Calmette-Guérin (BCG) scars or previous vaccination. Two patients developed tuberculosis after HSCT, the incidence of tuberculosis among those with positive QFT-GIT was 2.80 and among positive TST was 0 per 100 patients-years.^33^ Thus, further studies are needed to validate the use of QFT-GIT in HSCT patients.

## Conclusion

Most of reports of TB in HSCT patients were from ASIA, with TB incidence varying from 0. 0014 (USA) to 16% (Pakistan), and the lung was the organ most often involved. The mortality varied from 0 to 50%, and it was higher in allogeneic HSCT than autologous HSCT. Thus, TB is more frequent and with worse outcome among allogeneic HSCT. Risk factors associated with TB in allogeneic HSCT are use of steroid and GVHD. There is no consensus regarding the screening with either TST or QFT-GIT, use of primary prophylaxis for latent TB, and, whether in a developing country with high prevalence of TB, the epidemiologic query should be emphasized.

## Figures and Tables

**Table 1:** Clinical and radiological manifectation of tuberculosis in HSCT recipients

Study	Number of cases	Type of HSCT	HSCT conditioning regimen used	GVHD	Involved organs (number of patients)	Clinical manifestation (number of patients)	Radiological finding (number of patients)
Roy and Weisdorf. 1997.	2	Allogeneic (2)	not described (2)	yes (1)	Lung (1), central venous catheter (4), bone marrow (2), maxillary sinus (1),vertebral (1), bacteremia (3), skin (1), pleural effusion (1)	unexplained fever, pulmonary infiltrates, osteomyelitis or central venous catheter tunnel inflammation	not described (2)
Y-K Keung *et al.* 1998.	1	Autologous	TBI+ etoposide+ CFF	no	Lung	dyspnea, nonproductive cough, fever	bilateral Infiltrates in the upper and mid lungs
Ip, Yuen, Woo, *et al*. 1998.	10	Allogeneic (10)	TBI (9), no TBI (1)	yes (10)	Lung (10)	Cough (8), fever (9), left chest pain (1), dyspnea (1)	Cavity (2); right middle lobe infiltrate (1); right upper lobe infiltrate (1); right/left lower lobe infiltrate (1); Multiple infiltrate (1); Diffuse alveolar shadow (1) and normal (2).
M Aljurf *et al*. 1999.	4	Allogeneic (4)	Bu+CF(3), TBI + CF(1)	yes (3)	Spine (1), Lung (2), CNS(1)	fever (2), night sweats and a history of back pain (1), cough (2), dyspnea (1), seizure (1).	X-ray of the spine: destructive lesions and CT scan of the spine showed a large paravertebral abscess (1); Chest X-ray and CT scan showed areas of consolidation (1); Chest X-ray was normal and CT scan of the brain showed mild dilatation of the ventricular system.
de la Cámara *et al*. 2000.	20	Allogeneic (12)/Autologous(8)	BUCY (2), TBI+CF (9), CF+ATG (1)/CBV (3), Bu+CF (1), CCT(1), Melphalan (l), TBI+CF (1), BUCY (D)	yes (9)/no (8)	Knee (1), Lung (8), Pleura (1), Brain (1), Mng (1)/Lung (7) Oral ulcer (1)	fever (15), cough (13), constitutional syndrome (4), dyspnea (4), pleuntic chest pain (1), knee pain (1), oral pain (1) and seizures (2)	Normal chest X-ray (2), lung infiltrates (14), multiple and bilateral lesions (5)
Ku *et al*. 2001.	8	Allogeneic (8)	not described (2)	yes (7)	lung (8)	not described (2)	ches CT scan was normal alveolar Infiltrates (5)
B George *et al.* 2001.	3	Allogeneic (3)	Bu+CF (3)	yes (2)	Cervical spine, lymph node (1); Liver, bone marrow (1); Lung, liver, (1). bone marrow, spleen (1)	pain and swelling in the neck and fever (1); fever(2); pancytopenia	MRI scan: contiguous destruction of C7-D3 spine (1); not described (1); progressive pulmonary infiltrates (1).
Altclas *et al.* 2004.	1	Allogeneic	not described	yes	not described	lungs	alveolar Infiltrate in the right upper lobe
Lee *et al*. 2004.	9				Lung (8), pericardium (1)		
J-H Yoo *et al*.2005.	8	Allogeneic (7)	not described	yes (6)	not described	not described	not described
Garces Ambrossi *et al.* 2005.	4	Allogeneic (4)	TBI + CF(1), BU+CF(1), TBI+ATG+CF+ THIO(2)	yes (2)	lung (3), neck ymph node (l)	not described (1), mass in right neck (l), fever (2),cough (1)	chest X-ray showed a miliary pattern (1), chest CT scan showed a right upper lobe nodule (1), Chest X-ray showed bilateral upper lobe infiltrates (1), not described (1)
Al-Anazl *et al*. 2007.	3	Allogeneic (3)	TBI + CF (3)	yes (3)	Lung (3), liver and bone marrow (1).	Negative (2), reactivation of an old TB Infection before HSCT (1)	Negative (2), not described (1)
Machado CM, *et al*. 2009.	2	Allogeneic (2)	not described (2)	yes(2)	not described (2)	not described (2)	not described (2)
Costa SF, *et al*. 2010.	1	Allogeneic	Bu+CF	yes	lung	fever, chest pain, dyspnea, hypoxemia and pericarditis.	normal chest X-ray and Chest computed tomography (CT) scan showed tree-in-bud.
F-C Kuan, *et al*. 2011.	1	Autologous	not described	no	extrapulmonary	pancytopenia and maturation arrest of myeloid cells in the BM	normal chest X-ray

TBI: Total body Irradiation; CF: Cyclophosphamide; Bu: Bussulfan; CY: cyclosporine; CCT: cyclophosphamide, thlotepa, carboplatin; BEAM: BCNU, etoposide, Ara-C, melphalan; CBV: cyclophosphamide, BCNU, etoposide; THIO: thiotepa; CBV: cyclophosphamide, BCNU, etoposide; Mng: meningeal, BM; bone narrow;

**Table 2:** Diagnostic methods characteristics and reported sensitivity and specificity for general population. AFB= Acid Fast Bacilli

Method	Advantages	Disadvantages	Sensitivity	Specificity
AFB	Fast Low cost	Low sensitivity Differential diagnosis of AFB+	40–60%	95%
Culture	Specie identification Susceptibility tests	Delay in results Low sensitivity	60–80%	98%
Histology+	Shows tissue damage and granulomas with caseous necrosis that strongly supports a diagnosis	Invasive procedure it is not pathognomonic	60%	NA
Molecular/PCR	Fast result Species discrimination	Susceptibility tests Cost	78%	93%

* Data available for pleural and ganglionar involvement. NA. Non-available

**Table 3:** Recommended treatment schedule for TB for a patient with more than 110 lb.

	Therapeutic drugs	Duration
Induction phase	Rifampicin 600mg Isoniazid 300 mg Pyrazinamide 1600 mg Ethambutol 1200mg	2 months
Maintenance phase	Rifampicin 600mg Isoniazid 400mg	7–10 months ^*^ If disseminate disease or if chronic GVHD

## References

[1] Navari RM, Sullivan KM, Springmeyer SC (1983). Mycobacterial infections in marrow transplant patients. Transplantation.

[2] Akan H, Arslan O, Akan OA (2006). Tuberculosis in stem cell transplant patients. J Hosp Infect.

[3] Roy V, Weisdorf D (1997). Mycobacterial infections following bone marrow transplantation: a 20 year retrospective review. Bone Marrow Transplant.

[4] Keung YK, Nugent K, Jumper C, Cobos E (1999). Mycobacterium tuberculosis infection masquerading as diffuse alveolar hemorrhage after autologous stem cell transplant. Bone Marrow Transplant.

[5] Kurzrock R, Zander A, Vellekoop L, Kanojia M, Luna M, Dicke K (1984). Mycobacterial pulmonary infections after allogeneic bone marrow transplantation. Am J Med.

[6] de la Câmara R, Martino R, Granados E (2000). Tuberculosis after hematopoietic stem cell transplantation: incidence, clinical characteristics and outcome. Spanish Group on Infectious Complications in Hematopoietic Transplantation. Bone Marrow Transplant.

[7] Budak-Alpdogan T, Tangün Y, Kalayoglu-Besisik S (2000). The frequency of tuberculosis in adult allogeneic stem cell transplant recipients in Turkey. Biol Blood Marrow Transplant.

[8] Ip MS, Yuen KY, Woo PC (1998). Risk factors for pulmonary tuberculosis in bone marrow transplant recipients. Am J Respir Crit Care Med.

[9] Ku SC, Tang JL, Hsueh PR, Luh KT, Yu CJ, Yang PC (2001). Pulmonary tuberculosis in allogeneic hematopoietic stem cell transplantation. Bone Marrow Transplant.

[10] Wang J, Chang Y, Lee L (2004). Diffuse Pulmonary Infiltrates After Bone Marrow Transplantation: The Role of Open Lung Biopsy. Ann Thorac Surg.

[11] Cordonnier C, Martino R, Trabasso P, European Blood and Marrow Transplant Group Infectious Diseases Working Party (2004). Mycobacterial infection: a difficult and late diagnosis in stem cell transplant recipients. Clin Infect Dis.

[12] Aljurf M, Gyger M, Alrajhi A (1999). Mycobacterium tuberculosis infection in allogeneic bone marrow transplantation patients. Bone Marrow Transplant.

[13] Ullah K, Ahmed P, Raza S (2007). Allogeneic stem cell transplantation in hematological disorders: single center experience from Pakistan. Transplant Proc.

[14] Altclas J, Lescano A, Salgueira C (2005). Multidrug-resistant tuberculosis in bone marrow transplant recipient. Transpl Infect Dis.

[15] Maeda T, Kusumi E, Kami M, Tokyo Stem Cell Transplant (SCT) Consortium (2005). Disseminated tuberculosis following reduced-intensity cord blood transplantation for adult patients with hematological diseases. Bone Marrow Transplant.

[16] Khan B, Ahmed P, Ullah K, Hussain CA, Hussain I, Raza S (2005). Frequency of tuberculosis in haematological malignancies and stem cell transplant recipients. J Coll Physicians Surg Pak.

[17] Garces Ambrossi G, Jakubowski A, Feinstein MB, Weinstock DM (2005). Active tuberculosis limited to foreign-born patients after allogeneic hematopoietic stem cell transplant. Bone Marrow Transplant.

[18] George B, Mathews V, Srivastava A, Chandy M (2004). Infections among allogeneic bone marrow transplant recipients in India. Bone Marrow Transplant.

[19] Lee J, Lee MH, Kim WS (2004). Tuberculosis in hematopoietic stem cell transplant recipients in Korea. Int J Hematol.

[20] Erdstein AA, Daas P, Bradstock KF, Robinson T, Hertzberg MS (2004). Tuberculosis in allogeneic stem cell transplant recipients: still a problem in the 21st century. Transpl Infect Dis.

[21] Yoo JH, Lee DG, Choi SM (2004). Infectious complications and outcomes after allogeneic hematopoietic stem cell transplantation in Korea. Bone Marrow Transplant.

[22] Kerridge I, Ethell M, Potter M, Prentice HG (2003). Mycobacterium tuberculosis infection following allogeneic peripheral blood stem cell transplantation. Intern Med J.

[23] Kindler T, Schindel C, Brass U, Fischer T (2001). Fatal sepsis due to Mycobacterium tuberculosis after allogeneic bone marrow transplantation. Bone Marrow Transplantation.

[24] Campos A, Vaz CP, Campilho F (2000). Central nervous system (CNS) tuberculosis following allogeneic stem cell transplantation. Bone Marrow Transplant.

[25] Ullah K, Raza S, Ahmed P (2008). Post-transplant infections: single center experience from the developing world. Int J infect Dis.

[26] Machado CM, Martins TC, Colturato I (2009). Epidemiology of neglected tropical diseases in transplant recipients. Review of the literature and experience of a Brazilian HSCT center. Rev Inst Med Trop Sao Paulo.

[27] Russo RL, Dulley FL, Suganuma L (2010). Tuberculosis in hematopoietic stem cell transplant patients: case report and review of the literature. Int J Infect Dis.

[28] Kuan FC, Lin PY, Hwang CE (2011). Pancytopenia and myeloid maturation arrest in an autologous stem cell transplant recipient. Bone Marrow Transplant.

[29] Rouleau M, Senik A, Leroy E, Vernant JP (1993). Long-term persistence of transferred PPD-reactive T cells after allogeneic bone marrow transplantation. Transplantation.

[30] Ahmed P, Anwar M, Khan B (2005). Role of isoniazid prophylaxis for prevention of tuberculosis in haemopoietic stem cell transplant recipients. J Pak Med Assoc.

[31] Gauchon A, André N, Rome A (2008). Evaluation of a screening strategy after occurence of two simultaneous contaminating tuberculosis cases in a pediatric oncology department. Arch Pediatr.

[32] Tavil B, Gulhan B, Ozcelik U (2007). Tuberculin skin test positivity in pediatric allogeneic BMT recipients and donors in Turkey. Pediatr Transplant.

[33] Moon SM, Lee SO, Choi SH, Kim YS, Woo JH, Yoon DH, Suh C, Kim DY, Lee JH, Lee JH, Lee KH, Kim SH (2013). Comparison of the QuantiFERON-TB Gold In-Tube test with the tuberculin skin test for detecting latent tuberculosis infection prior to hematopoietic stem cell transplantation. Transpl Infect Dis.

[34] Keung YK, Nugent K, Jumper C (1999). Mycobacterium tuberculosis infection masquerading as diffuse alveolar hemorrhage after autologous stem cell trans- plant. Bone Marrow Transplant.

[35] Friedrich W, O’Reilly RJ, Koziner B (1982). T-lymphocyte reconstitution in recipients of bone marrow transplants with or without GVHD: imbalances on T-cell subpopulation having unique regulatory and cognitive function. Blood.

[36] Lum LG, Seigneuret MC, Orcutt-Thordarson N (1985). The regulation of immunoglobulin synthesis after HLA identical bone marrow transplantation. Blood.

[37] Al-Anazi KA, Al-Jasser AM, Evans DA (2007). Infections caused by mycobacterium tuberculosis in patients with hematological disorders and in recipients of hematopoietic stem cell transplant, a twelve year retrospective study. Ann Clin Microbiol Antimicrob.

[38] Jung JI, Lee DG, Kim YJ (2009). Pulmonary tuberculosis after hematopoietic stem cell transplantation: radiologic findings. J Thorac Imag.

[39] Delogu G, Herrmann JL (2012). Mycobacterium species. European Manual of Clinical Microbiology.

[40] Yamazaki R, Mori T, Nakazato T, Okamoto S (2010). Non-Tuberculous Mycobacterial Infection Localized in Small Intestine Developing after Allogeneic Bone Marrow Transplantation. Inter Med.

[41] Shi JM, Cai Z, Huang H, Ling MF (2009). Role of CT-guided percutaneous lung biopsy in diagnosis of pulmonary fungal infection in patients with hematologic diseases. Int J Hematol.

[42] Redelman-Sidi G, Sepkowitz KA (2013). Interferon Gamma Release Assays in the Diagnosis of Latent Tuberculosis Infection Among Immunocompromised Adults. Am J Respir Crit Care Med.

[43] Pai M, Palamountain KM (2012). New tuberculosis technologies: challenges for retooling and scale-up. Int J Tuberc Lung Dis.

[44] MMWR (2009). Updated Guidelines for the Use of Nucleic Acid Amplification Tests in the Diagnosis of Tuberculosis.

[45] Lee JI, Lee BJ, Roy EY (2011). The diagnostic accuracy of tuberculosis real-time polymerase chain reaction analysis of computed tomography-guided bronchial wash samples. Diagn Microb Infec Dis.

[46] Min J-W, Yoon HI, Park KU (2010). Real-time polymerase chain reaction in bronchial aspirate for rapid detection of sputum smear-negative tuberculosis. Int J Tuberc Lung Dis.

[47] Abubakar I, Griffiths C, Ormerod P (2012). Diagnosis of active and latent tuberculosis: summary of NICE guidance. BMJ.

[48] Horne DJ, Narita M, Spitters CL (2013). Challenging Issues in Tuberculosis in Solid Organ Transplantation. Clin Infect Dis.

[49] Lawn SD, Mwaba P, Bates M (2013). Advances in tuberculosis diagnostics: the Xpert MTB/RIF assay and future prospects for a point-of-care test. Lancet Infect Dis.

[50] Rajpal S, Kashyap RS, Nayak AR (2013). Laboratory Investigations on the Diagnosis of Tuberculosis in the Malnourished Tribal Population of Melghat, India. Plos One.

[51] Dubey A, Gwal R, Agrawal S (2013). Mycobacterium tuberculosis detection in blood using multiplex nested polymerase chain reaction. Int J Tuberc Lung Dis.

[52] Bento J, Silva AS, Rodrigues F, Duarte R (2011). Métodos Diagnósticos em Tuberculose. Acta Med Port.

[53] Hopewell PC, Pai M, Maher D (2006). International standards for tuberculosis care. Lancet Infect Dis.

[54] Yew WW, Lange C, Leung CC (2011). Treament of tuberculosis: update 2010. Eur Respir J.

[55] World Health Organization (2009). Treatment of Tuberculosis Guidelines.

[56] American Thoracic Society: CDC; Council of the Infectious Disease Society of America (2000). Diagnostic standards and classification of tuberculosis in adults and children. Am J Respir Crit Care Med.

[57] Ministério da Saúde do Brasil http://portal.saude.gov.br/portal/arquivos/pdf/nota_tecnica_versao_28_de_agosto_v_5.pdf.

[58] La Camara R, Martino R, Granados E (2000). Tuberculosis after hematopoietic stem cell transplantation: incidence, clinical characteristics and outcome. Bone Marrow Transplantation.

[59] Maeda T, Kusumi E, Kami M (2005). Disseminated tuberculosis following reduced-intensity cord blood transplantation for adult patients with hematological diseases. Bone Marrow Transplantation.

[60] De Assis RA, Kerbauy FR, Rodrigues M (2009). Mycobacterium tuberculosis infection: a rare late complication after cord blood hematopoietic SCT. Bone Marrow Transplantation.

[61] Aguado JM, Torre-Cisneros J, Munoz P (2009). Tuberculosis in Solid-Organ Transplant Recipients: Consensus Statement of the Group for the Study of Infection in Transplant Recipients (GESITRA) of the Spanish Society of Infectious Diseases and Clinical Microbiology. Clin Infect Dis.

[62] Saukkonen JJ, Cohn DL, Jasmer RM, ATS (American Thoracic Society) Hepatotoxicity of Antituberculosis Therapy Subcommittee (2006). An official ATS statement: hepatotoxicity of antituberculosis therapy. Am J Respir Crit Care Med.

[63] Devarbhavi H (2012). Adaptation and Antituberculosis Drug–induced Liver Injury. Am J Respir Crit Car Med.

[64] Altclas J, Lescano A, Palmero D (2005). Multidrug-resistant tuberculosis in bone marrow transplant recipient. Transpl Infect Dis.

[65] Lienhardt C, Raviglione M, Spigelman M (2012). New Drugs for the Treatment of Tuberculosis: Needs, Challenges, Promise, and Prospects for the Future. J Infect Dis.

[66] Uhlin M, Andersson J, Zumla A, Maeurer M (2012). Adjunct Immunotherapies for Tuberculosis. J Infec Dis.

